# Long-Term Epidemiological Dynamics of Japanese Encephalitis Infection in Gansu Province, China: A Spatial and Temporal Analysis

**DOI:** 10.4269/ajtmh.20-0179

**Published:** 2020-09-28

**Authors:** Xuxia Wang, Li Su, Hongwen Zhu, Wenbiao Hu, Jing An, Caixia Wang, Qiannan E, Xin Qi, Guihua Zhuang

**Affiliations:** 1 School of Public Health, Xi’an Jiaotong University Health Science Center, Xi’an, China;; 2 Health Hotline, Gansu Provincial Center for Disease Control and Prevention, Lanzhou, China;; 3 School of Public Health, Lanzhou University, Lanzhou, China;; 4 Lanzhou University Second Hospital, Lanzhou, China;; 5 School of Public Health and Social Work, Queensland University of Technology, Kelvin Grove, QLD, Australia

## Abstract

The incidence of Japanese encephalitis (JE) has greatly declined in China. However, JE incidence has significantly increased in Gansu in recent years, on the top of ranks among all provinces in China. To explore the spatial spread and resurgence of JE transmission in Gansu in the past 60 years, we collected yearly data on reported JE in each county (1958–2017) and monthly data on JE cases (1968–2017), respectively. We grouped the dataset into six categories, each consisting of a 10-year period between 1958 and 2017. Spatial cluster analysis was applied to identify the potential space–time clusters of JE incidence, and logistic regression models were used to identify the spatial and temporal dispersion of JE. Japanese encephalitis incidence in Gansu showed an upward trend from 1970 to 1977 and peaked in 1974, then declined, and fluctuated over the study period until an outbreak again in 2017. Japanese encephalitis incidence for the first 30-year period (1958–1987) peaked in September each year and thereafter peaked in July and August during 1988–2017. Spatial cluster analysis showed the geographical range of JE transmission fluctuated over the past 60 years. The high-incidence clusters of JE were primarily concentrated in the southeast of Gansu. We found significant space–time clustering characteristics of JE in Gansu, and the geographical range of notified JE cases has significantly expanded over recent years. The potential rebound of JE transmission occurred in 2016–2017 should be placed on the top priority of government work during the control and prevention of JE in Gansu, China.

## INTRODUCTION

Japanese encephalitis (JE), caused by JE virus (JEV), is an acute infectious zoonosis disease transmitted by a *Culex* mosquito spp. and is the most common cause of encephalitis in Asia.^1–3^ There are significant JE burdens in 24 countries in Asian and Western Pacific regions. Approximately 3 billion people live in JE-endemic areas, with 67,900 JE cases occurring annually (overall incidence: 1.8 per 100,000). Of these, only 50% occur in China and about 10% are reported to the WHO.^4^ The fatality rate among JE patients is approximately 20–30%, and, meanwhile, 30–50% of the survivors exhibit significant neurological sequelae.^5,6^

Japanese encephalitis experienced two devastating epidemics in the 1960s and 1970s.^5,7,8^ Since the 1980s, the epidemics have been substantially reduced because of the use of commercial JE vaccines among children.^9^ At the end of 2007, the JE vaccines were included in China’s National Expanded Program on Immunization, resulting in a high rate for vaccination among children, except for the western non-endemic provinces (including Qinghai, Tibet, and Xinjiang).^10^ After that, reported JE cases also dramatically decreased to less than 5,000 per year in China according to the National Notifiable Disease Reporting System (NNDRS).^11^

In general, Gansu Province is a medium–low endemic area of JE in China,^12–14^ and the annual incidence of JE was significantly lower than the national average during 1958–2012.^15^ Our previous studies suggested that the adults’ incidence (≥ 45 years) of JE cases increased between 2002 and 2008 in Gansu Province,^16^ and most JE cases were located in the Tianshui, Longnan, and Pingliang municipalities, which lies in the southeast of Gansu.^17^ However, Gansu became the predominant source of JE cases reported in 2017, and accounted for 31.6% of the cases in Mainland China according to the NNDRS, especially three JE cases were reported in the western of Gansu. Therefore, the high incidence of JE among population has become a significant public health issue in Gansu in recent years and should not be ignored.

Characteristic description of spatial and temporal distribution is a time-efficient way to work out some effective measurements for JE control and prevention. Currently, several studies have explored the spatiotemporal patterns of JE cases in Nepal during 2007–2015^18^ and in China during 1965–1975 and 2002–2014.^8,19,20^ However, methods of spatiotemporal analysis were different in these studies, and, meanwhile, few studies focused on the long time-scale reappearance of JE over the past few decades, especially in rural regions in China. This study aims to identify the potential space–time clusters of reported JE cases for the 60-year period, and explore the spatial and temporal dispersion of JE in Gansu and, hence, to provide evidence-based suggestions for local policy-makers and service providers for disease control and prevention.

## METHODS

### Study area.

**Figure 1.**
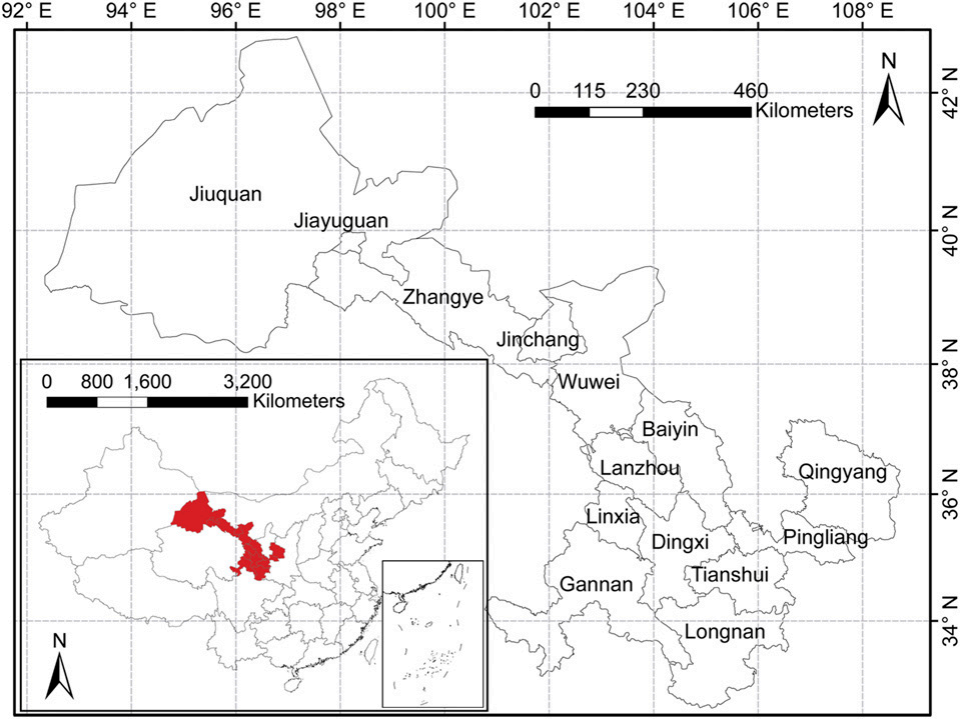
Geographical administrative division at the municipality level, Gansu Province, China. The study areas and their location in Gansu Province and China. This figure is generated using ArcGIS software version 10.2 (ESRI). This figure appears in color at www.ajtmh.org.

Gansu Province is located in the northwest of China with latitude between 32°11′N and 42°57′N and longitude between 92°13′E and 108°46′E (Figure 1). The terrain of the study area is a narrow strip, situating in the border of the Loess Plateau, the Qinghai-Tibet Plateau, and the Inner Mongolia Plateau, occupying an area of 425.9 thousand square kilometers with 26.26 million permanent residents in 2018 (Gansu yearbook 2018).

### Data collection.

Japanese encephalitis cases are diagnosed by clinician and reported to the Chinese CDC. In 1958, JE was discovered for the first time in Gansu. Annual JE cases in each county (1958–2017) and monthly provincial JE cases (1968–2017) were obtained from the Gansu Provincial CDC and NNDRS, respectively; Annual JE incidence in each province in China (1958–2017) was obtained from the National Scientific Data Sharing Platform for Population and Health. Geographical changes of administrative division and district boundary of each county were collected from the Bureau of Statistics and Department of Civil Affairs of Gansu. Each county in Gansu corresponds to the county in the map of 2017 after adjusting for geographical boundary changes. If the administrative division of one county was split or merged, then the incidence of split or merged counties for specific years will be replaced by the average incidence of all related counties. This study was divided into six categories, including 1958–1967, 1968–1977, 1978–1987, 1988–1997, 1998–2007, and 2008–2017, based on change of epidemic from the relative higher risk period (1970–1977) to lower risk after 1978. Japanese encephalitis incidence at the county level was mapped using ArcGIS software version 10.2 (ESRI, Redlands, CA) to display its spatial distribution.

### Data analysis.

#### Spatiotemporal patterns using heatmap format.

In descriptive analysis, spatial trends are an important factor related to historical events and geographical features besides mapping spatial pattern of disease. Heatmaps are often used to represent variable data, including spatiotemporal factors such as gene expression experiments and ecological studies.^21–23^ Hence, we used the heatmap format to visualize the spatiotemporal profiles of JE annual incidence by ggplot2 function using R 3.6.1 software (RStudio, Boston, MA).^24^

#### Spatial smoothing using empirical Bayesian analysis.

Bayesian spatial smoothing was used to reduce random variation related to small populations.^25,26^ The method can allow identification of spatial disease clusters that may not be apparent from direct observation of the raw data.^25^ We grouped the dataset into six categories, each consisting of a 10-year period between 1958 and 2017. For each period, we spatially smoothed the JE incidence data by an empirical Bayesian spatial smoothing procedure using GeoDa software (version 1.12, Center for Spatial Data Science University of Chicago, Chicago, IL).^27^

#### Time series seasonal decomposition.

We performed the seasonal decomposition of time series by Loess (STL) function using R 3.6.1 tidyverse package during 1968–1977, 1978–1987, 1988–1997, 1998–2007, and 2008–2017.^28^ Time series were decomposed into three components: trend, seasonal, and remainder (residual). Seasonal decomposition of time series by Loess decomposition data were graphed on four panels: data (monthly JE incidence), trend (variation in the data in the long-term period), seasonal (variation in the data within a year), and remainder (variation that remains after removing seasonal and trend components).^29,30^

#### Spatial and temporal cluster analysis.

To evaluate high-risk space–time clusters of JE, we scanned for clusters with high rates using SaTScan software (version 9.4.2, Harvard Medical School and Harvard Pilgrim Health Care Institute, Boston, MA),^31^ and the discrete Poisson model was used to fit the spatial clusters of the six-category dataset.^19,32,33^ According to the fitted result of the discrete Poisson model, we used a maximum temporal cluster size of 50% of the study period in the temporal window and the maximum spatial cluster size of less than 10% of the total population at risk in the spatial window to identify space–time clusters. Also, the primary cluster and secondary clusters were detected through the log-likelihood ratio (LLR) test, and the significance of the clusters was evaluated by Monte Carlo simulation.

#### Dynamic spatial and temporal dispersion.

We attempted to identify whether changes in JE incidence varied with latitude and longitude of county centroids in the periods 1988–1997, 1998–2007, and 2008–2017. Logistic regression models were constructed with the dichotomous outcome variable defined as whether or not an increase of JE occurred in each county between the three periods. Longitude and latitude of county centroids were entered as explanatory variables. Spatial dispersions were expressed in terms of odds ratios (OR) for longitude and latitude, with 95% CIs.

## RESULTS

### Japanese encephalitis epidemics.

**Figure 2.**
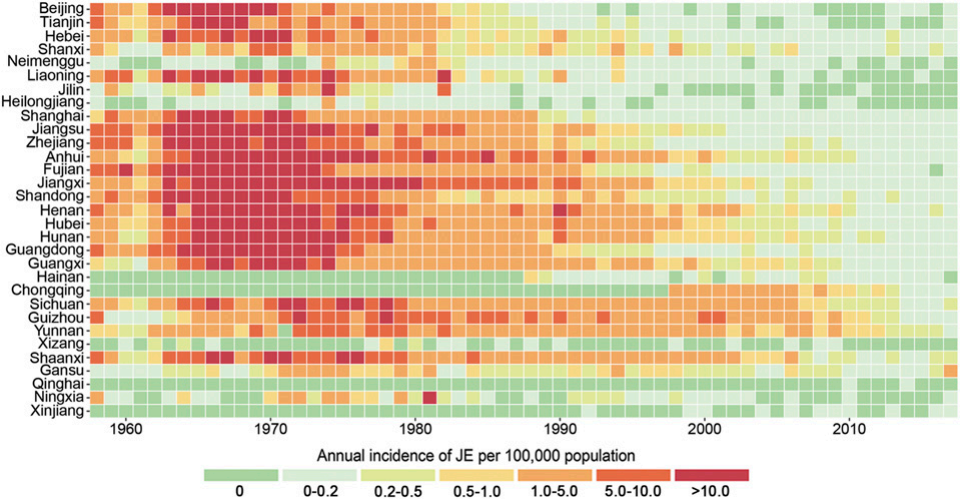
Heatmap representing annual Japanese encephalitis (JE) incidence of each province in Mainland China. The horizontal dimension indicates the year of JE infection, whereas the vertical size indicates the distribution of the epidemic in the province of Mainland China. The color of the cells represents the magnitude of the incidence of JE in that cell. This figure appears in color at www.ajtmh.org.

From 1958 to 2017, a total of 5,984 JE cases were reported in Gansu, with a cumulative average incidence of 0.48 per 100,000. There was a higher JE incidence rate between 1970 and 1977. From 1978 to 20,145, the incidence of JE cases in Gansu was at a relative low level and slightly fluctuated (Supplemental Figure). During 2016–2017, the incidence of JE showed a significant upward trend in Gansu, ranging from 0.32 per 100,000 to 1.39 per 100,000 and ranking the first in China, which were higher than the national average of 0.09 per 100,000 (2016) and 0.08 per 100,000 (2017), especially the outbreak in 2017 (Figure 2).

### Geographic distribution.

**Figure 3.**
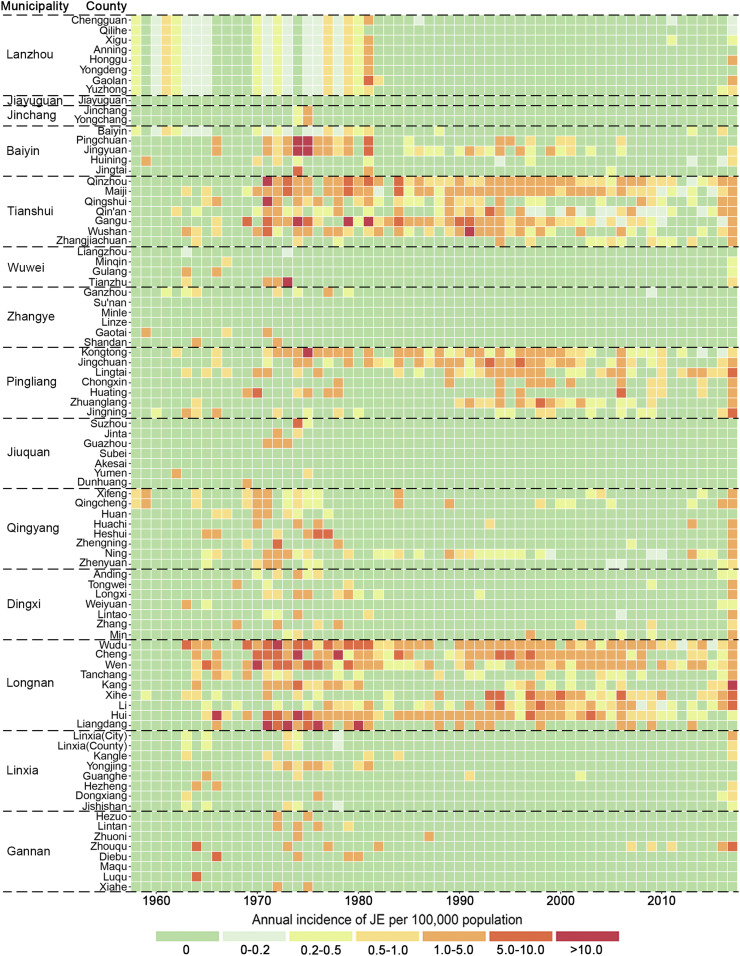
Heatmap representing annual Japanese encephalitis (JE) incidence of each county in Gansu Province, China. The horizontal dimension indicates the year of JE infection, whereas the vertical size indicates the distribution of the epidemic in county of Gansu. The color of the cells represents the magnitude of the incidence of JE in that cell. This figure appears in color at www.ajtmh.org.

**Figure 4.**
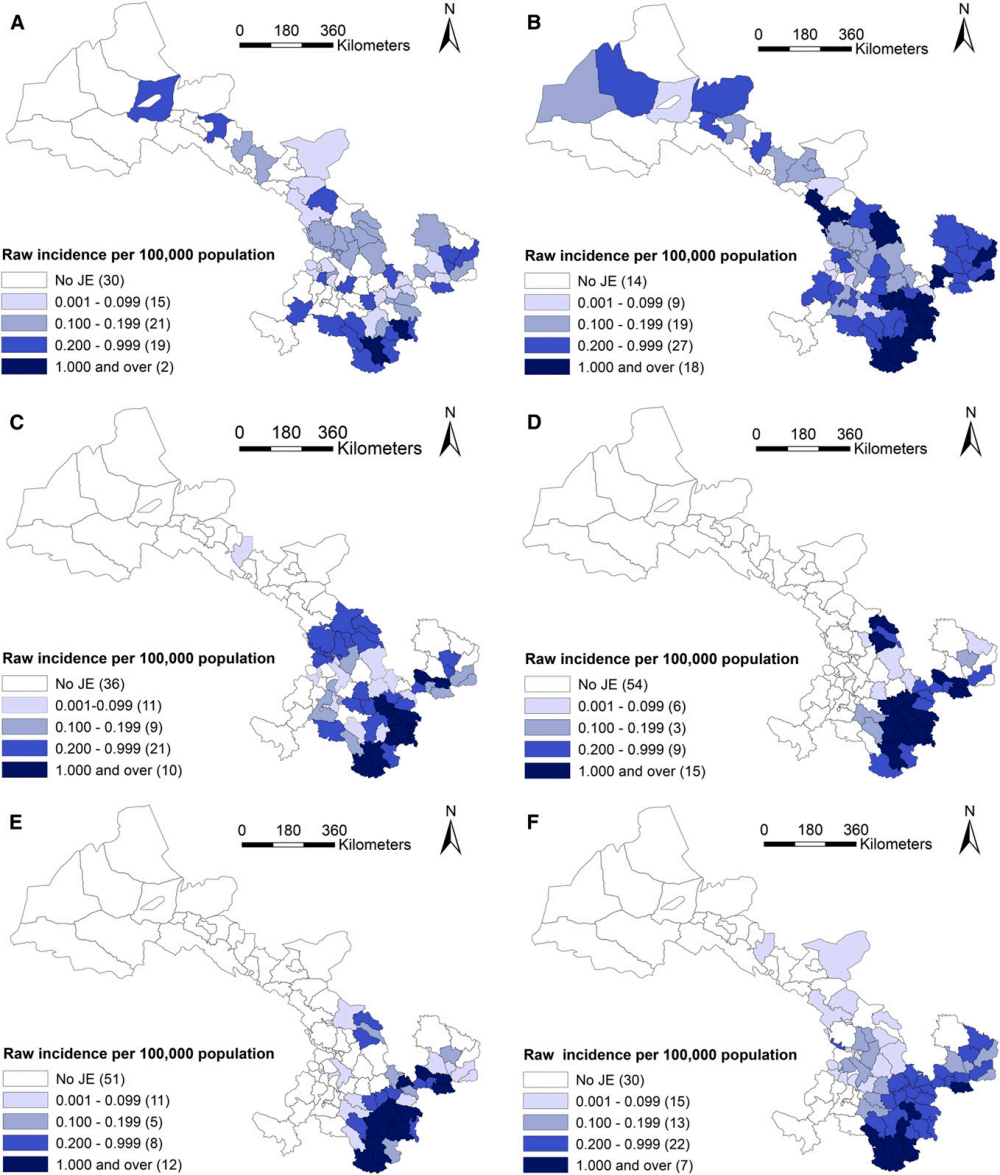
Raw incidence of Japanese encephalitis (JE) in six study periods in Gansu Province, China. (**A**) 1958–1967, (**B**) 1968–1977, (**C**) 1978–1987, (**D**) 1988–1997, (**E**) 1998–2007, and (**F**) 2008–2017. This figure is generated using ArcGIS software version 10.2 (ESRI). Mining areas in Gansu are not included in the map presented in this study, and no JE cases have been found in the areas. This figure appears in color at www.ajtmh.org.

According to administrative division in 2010, JE cases had been reported from 80 of 87 counties in Gansu, and seven counties had no cases reported. The geographical distribution of JE cases varies among the six periods, including 1958–1967, 1968–1977, 1978–1987, 1988–1997, 1998–2007, and 2008–2017, 10 years for each, based on the temporal variation of JE incidence in the whole province shown in Supplemental Figure. In Figures 3 and 4, JE cases in 1958–1967 and 1968–1977 dispersed throughout the province, with exception of seven counties (Jiayuguan, Sunan, Subei, Minle, Linze, Akesai, and Maqu). After that, JE cases were gradually concentrated in the southeastern area of Gansu from 1978 to 2007, and its prevalence regions were distributed in 51, 33 and 36 counties during 1978–1987, 1988–1997, and 1997–2007, respectively. At that time, Tianshui and Longnan municipalities, especially Maiji, Qinzhou, Gangu, and Wudu counties, were the regions with high JE incidence, which accounted for 74.3% (2,521/3,394) of total JE cases in Gansu. Then, the number of JE prevalence regions increased again during 2008–2017 and distributed in southeastern and central Gansu including 57 counties.

The highest annual incidence (per 100,000) in each period was reported in Hui (1.62) during 1958–1967, in Liangdang (7.36) during 1968–1977, in Gangu (4.16) during 1978–1987, in Cheng (3.83) during 1988–1997, in Xihe (2.80) during 1998–2007, and in Zhouqu (1.26) during 2008–2017.

**Figure 5.**
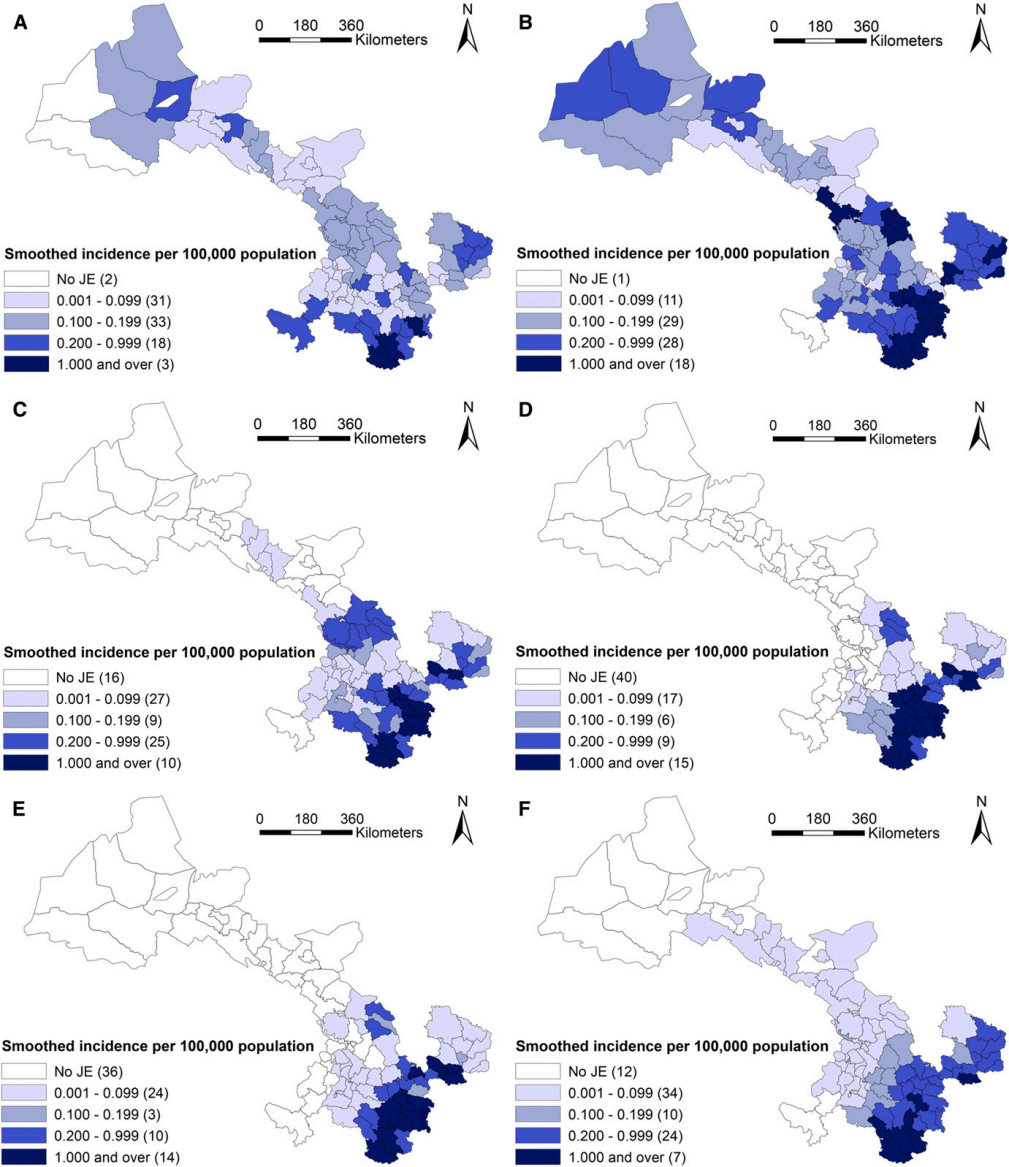
Smoothed incidence of Japanese encephalitis (JE) in six study periods in Gansu Province, China. (**A**) 1958–1967, (**B**) 1968–1977, (**C**) 1978–1987, (**D**) 1988–1997, (**E**) 1998–2007, and (**F**) 2008–2017. This figure is generated using ArcGIS software version 10.2 (ESRI, USA). Mining areas in Gansu are not included in the map presented in this study, and no JE cases have been found in the areas. This figure appears in color at www.ajtmh.org.

Furthermore, the geographic distribution of smoothed estimates of JE incidence in Gansu in the six time periods is depicted in Figure 5. It shows that the incidence of JE varied geographically across the province. Usually, a county with a small population (e.g., less than 1,000) at risk tended to have its observed rates adjusted considerably toward the neighborhood average. In Gansu, each county has considerable population (1,696 at least) during the whole study period. Thus the rate did not substantially change compared with the raw incidence in Figure 4.

### Seasonal pattern of JE cases.

**Figure 6.**
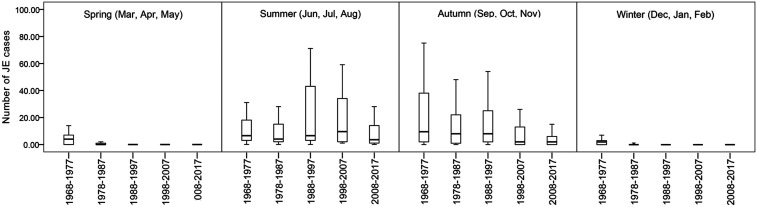
Boxplots of monthly Japanese encephalitis cases across four seasons during the five study periods in Gansu Province, China (1968–1977, 1978–1987, 1988–1997, 1998–2007, and 2008–2017). The top and bottom of the box indicate the upper quartile (P75) and the lower quartile (P25), respectively; the line in the middle of the box represents the median value; and the top and bottom lines are minimum and maximum, respectively.

**Figure 7.**
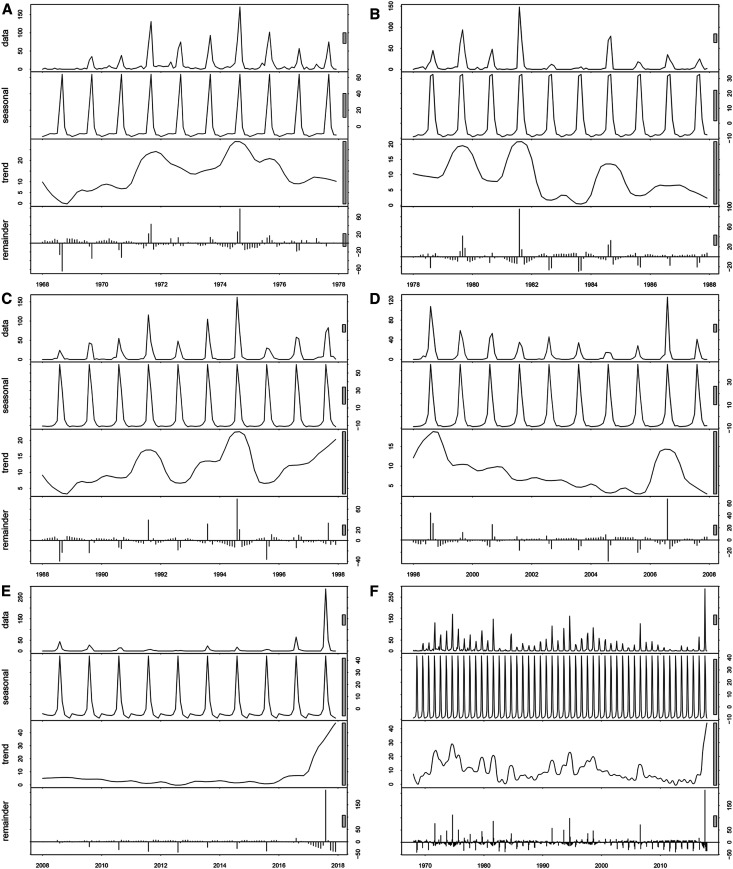
Trend, and seasonal and residual (remainder) components derived from the seasonal decomposition of time series by Loess decomposition of monthly Japanese encephalitis case, during the five study periods in Gansu Province, China. (**A**) 1968–1977, (**B**) 1978–1987, (**C**) 1988–1997, (**D**) 1998–2007, and (**E**) 2008–2017.

The distribution of JE cases in Gansu showed obvious seasonal patterns of occurrence. Over the study period, the general trend of JE cases was an initial increase from July followed by a peak in September or in August, respectively (Figures 6 and 7). During 1968–1987, the seasonal distribution of JE cases showed that autumn was the peak seasons for JE, followed by summer. After that, the incidence of JE in summer gradually exceeded in autumn from 1988 to 2017 (Figure 6). Figure 7 presents the monthly cases, trend, and seasonal and residual (remainder) components derived from seasonal-trend decomposition. The STL decomposition showed that seasonality is the most important component during the all-study period, and the incidence has shown more regular seasonal characteristics since 1988. These results suggest that the seasonal pattern epidemic transmission peak gradually moved ahead since 1988.

### Spatial cluster analysis.

**Figure 8.**
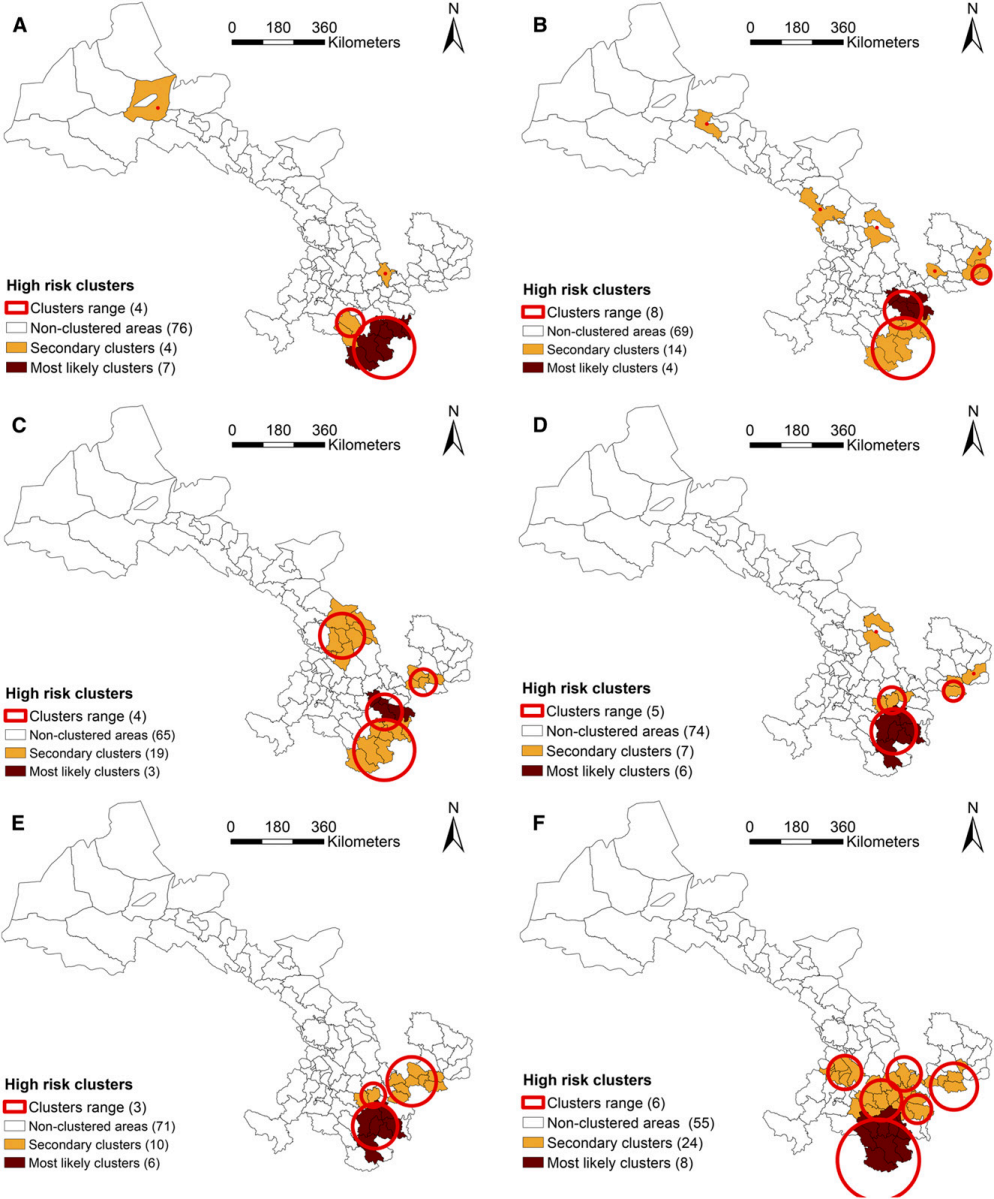
The clusters of Japanese encephalitis (JE) cases in Gansu Province in China, in six study periods. (**A**) 1958–1967, (**B**) 1968–1977, (**C**) 1978–1987, (**D**) 1988–1997, (**E**) 1998–2007, and (**F**) 2008–2017. This figure is generated using ArcGIS software version 10.2 (ESRI). Mining areas in Gansu are not included in the map presented in this study, and no JE cases have been found in the areas. This figure appears in color at www.ajtmh.org.

The cluster analyses showed that the space–time distribution of JE was clustered during six periods: 1958–1967, 1968–1977, 1978–1987, 1988–1997, 1998–2007, and 2008–2017. Using the maximum spatial cluster size of 10% of the population at risk, the primary cluster was identified in all six periods, mainly located in southeastern Gansu (Figure 8).

The specific information including the name of counties, radius (km), observed cases, expected cases, RR, and LLR in each cluster is listed in Table 1. During 1958–1967, the primary cluster was in the south of Gansu and was composed of the seven counties (RR = 15.85), and four secondary clusters were discovered (RR: 9.39–27.52). The JE-clustered areas substantially increased from 1968 to 1987 compared with the previous 10 years. From 1988 to 2007, the proportion of counties in clusters decreased, compared with earlier years in this study. In the most recent decade of the study (2008–2017), eight counties were identified as the primary cluster of JE (RR = 18.64), and five secondary clusters were discovered (RR: 3.50–16.04). In summary of these results, Wudu, Cheng, Li, Hui, and Xihe, which were economically backward areas, were the clusters for JE in all periods.

**Table 1:** Clusters of Japanese encephalitis cases, Gansu Province, China, in six different periods (1958–1967, 1968–1977, 1978–1987, 1988–1997, 1998–2007, and 2008–2017)

Cluster	Name of counties	Radius (km)	Time frame	Number of observed cases	Number of expected cases	RR	Log-likelihood ratio*
1958–1967							
1†	Kang, Wudu, Cheng, Xihe, Hui, Wen, and Liangdang	98.81	1963–1966	83	7.84	15.85	134.83
2	Jingning	0	1966	13	0.50	27.52	30.22
3	Tanchang and Zhouqu	43.85	1964–1966	11	1.22	9.39	14.60
4	Yumen	0	1962	5	0.23	22.03	10.64
1968–1977							
1†	Gangu, Qingshui, Qinzhou, and Maiji	59.91	1971–1975	390	66.19	7.41	403.59
2	Kang, Wudu, Cheng, Xihe, Hui, Wen, and Liangdang	98.81	1970–1974	324	66.13	5.85	279.26
3	Jingyuan	0	1974–1977	123	15.51	8.49	150.86
4	Kongtong	0	1975–1976	42	6.43	6.68	43.63
5	Ning and Zhengning	29.27	1972–1973	40	9.68	4.21	26.71
6	Tianzhu	0	1973	16	1.51	10.66	23.30
7	Suzhou	0	1974	18	2.42	7.52	20.63
8	Heshui	0	1976–1977	15	2.29	6.60	15.53
1978–1987							
1†	Qinzhou, Maiji, and Gangu	57.06	1978–1981	216	27.08	9.80	278.34
2	Kang, Wudu, Cheng, Xihe, Hui, Wen, and Liangdang	98.81	1978–1981	204	32.04	7.67	221.20
3	Baiyin, Gaolan, Jingyuan, Chengguan, Yuzhong, Jingtai, Anning, and Pingchuan	69.79	1981	57	9.93	6.02	53.63
4	Chongxin, Huating, Kongtong, and Jingchuan	41.60	1978–1981	46	17.50	2.70	16.36
1988–1997							
1†	Xihe, Li, Cheng, Qinzhou, Hui, and Wudu	72.04	1993–1997	364	69.41	6.81	345.34
2	Gangu, Wushan, and Qin’an	41.96	1990–1994	251	41.16	7.26	261.72
3	Lingtai and Jingchuan	31.23	1993–1997	94	15.66	6.38	92.47
4	Ning	0	1989	17	2.57	6.68	17.76
5	Jingyuan	0	1996	16	2.56	6.32	15.97
1998–2007							
1†	Xihe, Li, Cheng, Qinzhou, Hui, and Wudu	72.04	1998–2002	316	46.95	9.52	377.35
2	Huating, Chongxin, Kongtong, Zhangjiachuan, Zhuanglang, Qingshui, Lingtai, and Jingchuan	77.45	1998	65	8.47	8.16	77.63
3	Gangu and Wushan	38.24	1998–1999	22	7.29	3.06	9.70
2008–2017							
1†	Wen, Wudu, Zhouqu, Kang, Xihe, Cheng, Tanchang, and Li	132.55	2017	110	6.92	18.64	209.14
2	Jingning, Zhuanglang, Tongwei, and Qin’an	53.53	2017	74	5.12	16.04	132.30
3	Maiji, Qingshui, and Qinzhou	45.18	2016–2017	60	8.12	7.98	70.07
4	Lingtai, Jingchuan, Chongxin, Xifeng, and Huating	73.88	2017	36	3.10	12.18	56.14
5	Wushan, Gangu, Zhang, Longxi, and Min	64.73	2017	30	5.79	5.36	25.55
6	Guanghe, Kangle, Lintao, Dongxiang, Hezheng, Linxia (city), and Linxia (county)	53.12	2017	19	5.53	3.50	10.10

* *P* < 0.05.

† Primary cluster.

Spatiotemporally, the number of the primary clustered counties decreased over time, except increases in 1988–1997 and in 2008–2017, and, meanwhile, the location of secondary clusters (mainly located in southeastern and central regions in Gansu) also varied over time. In addition, the secondary clusters were first identified in Linxia municipality in the central south of Gansu during 2017, including six counties.

### Spatiotemporal dispersion.

Table 2 indicated that changes of JE were significantly associated with longitude (OR = 1.25, 95% CI: 1.05–1.50) between the periods 2008–2017 and 1988–1997, associated with longitude (OR = 1.67, 95% CI: 1.21–2.30) and latitude (OR = 0.55, 95% CI: 0.35–0.87) between the periods 1998–2007 and 1988–1997, and associated with longitude (OR = 1.34, 95% CI: 1.10–1.63) between the periods 2008–2017 and 1998–2007.

**Table 2:** Changes of Japanese encephalitis on latitude and longitude, Gansu Province, China, 1988–2017

Changes in periods*	Longitude		Latitude	
	OR	95% CI	OR	95% CI
Period 3–period 1	1.25	1.05–1.50	0.82	0.64–1.04
Period 2–period 1	1.67	1.21–2.30	0.55	0.35–0.87
Period 3–period 2	1.34	1.10–1.63	0.80	0.63–1.02

OR = odds ratio.

* Period 1 = 1988–1997; period 2 = 1998–2007; period 3 = 2008–2017.

**Figure 9.**
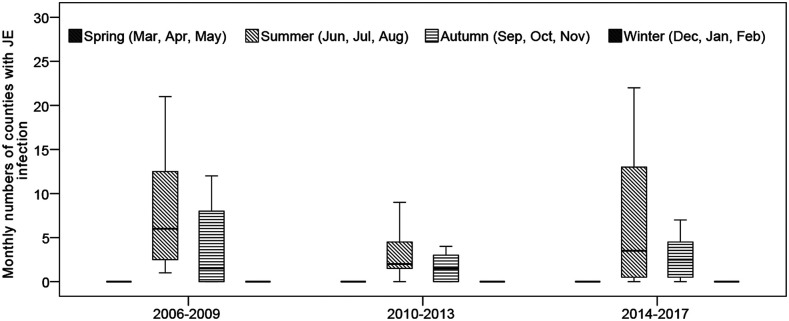
Boxplots of monthly numbers of counties with Japanese encephalitis infection across four seasons during the three study periods in Gansu Province, China (2006–2009, 2010–2013, and 2014–2017). The top and bottom of the box indicate the upper quartile (P75) and the lower quartile (P25), respectively; the line in the middle of the box represents the median value; the top and bottom lines are minimum and maximum, respectively.

Moreover, there was a striking variation in monthly numbers of counties with JE from 2006 to 2017, as shown in Figure 9. Boxplots of the monthly numbers of county with JE suggested that a downward trend of JE incidence occurred from 2006 to 2013, and after that an upward trend of JE incidence occurred in Gansu. Figure 9 also indicated a strongly seasonal pattern (with a peak in summer) and two large peaks of JE incidence occurred in summer during 2006–2009 (95 cases) and 2014–2017 (107 cases), respectively. Peaks in incident cases generally coincided with high monthly numbers of county with JE cases.

## DISCUSSION

In this study, we identified spatiotemporal characteristics and spatial clusters of JE in 1958–2017 in Gansu, China, using laboratory-confirmed and clinically diagnosed JE cases from the NNDRS. In the 1960s and 1970s, a devastating epidemic occurred in most provinces in China. Because of the Cultural Revolution in China at that time, the public health authorities were weakened in healthcare, environmental, and sanitary interventions on infectious disease control and prevention.^8^ Meanwhile, because JE vaccine had not yet begun to be applied, China experienced a natural JE epidemic period with no interventions.^11^ Compared with other provinces in China, there is no large-scale epidemic of JE in Gansu Province, and the epidemic peak lags other provinces, appearing in 1970s–1980s. This may be because of the higher altitude, lower temperature and rainfall, lower population density, and the livestock-based agriculture with relatively sparse pigsties and rice paddies, the breeding of mosquitoes, and the circulation of JEV would be hindered.^34^ In the 1980s, with the use of JE vaccine, the provincial average incidence of JE in Gansu gradually declined. However, the JE vaccine in Gansu has not yet been included in the immunization program, and, thus, children received the JE vaccination at their own expenses in Gansu. The traditional epidemic regions were poorly populated with insufficient mosquito prevention facilities. Meanwhile, local residents lacked anti-mosquito awareness and would not also come forward for JE vaccinations. Therefore, JE incidences had not yet been effectively controlled in these epidemic regions during 1978–2007. At the end of 2007, JE vaccine was included in the National Expanded Immunization Program in China, and the costs of vaccines and syringes were supplied by the central government. Thereafter, the incidence rate further fell to 0.02–0.20 per 100,000 for the period of 2009–2015 in Gansu, which reached the lowest level in history, suggesting an obvious decrease in JE after implementing the expanded immunization program.^10^

However, the incidence of JE in Gansu increased significantly in 2016 and 2017, which is higher than the national average level, and the epidemic transmission areas also showed an expanding trend. According to data from the NNDRS, the sudden increase in reported cases was mainly accompanied with an increase in adult infection. The number of adult JE cases over the age of 15 years accounted for 90.4% (in 2016) and 95.3% (in 2017) of the total JE cases in Gansu. There are evidences that childhood cases have been greatly reduced by immunization with vaccines, and then the age distribution of cases has shifted toward adults in some areas of Mainland and Taiwan of China, especially in the elderly,^9,35^ which is consistent with the present study. Currently, adult infection has also become a key issue for the prevention and control of JE in Gansu.^9,13^

Climate factors, especially temperature and precipitation, have been reported to be associated with the density of mosquitoes and are also the main drivers of JE.^36,37^ In the first decade of the epidemic records, however, the epidemic did not show obvious seasonal features, probably because the reporting system was still incomplete and there were underreported, delayed, and misdiagnosed cases, which partly masked the distribution characteristics of the epidemic. After the first decade, the occurrence of JE cases showed significant seasonal characteristics in Gansu and had an initial increase from July followed by a peak in September (1968–1987) or in August (1988–2017), respectively, and then declined thereafter. Li et al.^13^ reported that the peaked incidence of JE cases occurred from July to September in China during 2004–2014, which is in accordance with the present study. Similar studies reporting seasonal patterns of JE in Nepal (2004–2009) also indicated that JE cases increase from July followed by a peak in August, and subsequently decrease.^36,38^ Hence, the annually preventive measures of JE transmission in Gansu should be taken by relevant departments in advance.

Spatiotemporal cluster analysis is a valuable tool to examine how spatial patterns of disease change over time. This study showed that JE transmission in Gansu was clustered in six periods and provided a clear pattern of JE clustering within this province. During the study period, JE clustered mostly in the counties under the jurisdiction of Longnan municipality (including Wudu, Cheng, Li, and Xihe counties), which lies in the only subtropical climate region in Gansu. Besides, Longnan is adjacent to Sichuan Province, a region with high JE incidence in China. Counties in the primary cluster for JE transmission have high precipitation and mosquito density, clearly indicating that JE could be easily transmitted in these regions.^39–42^ Moreover, spatiotemporal distribution of secondary clusters showed a significant variation in the last 60 years. Most of the secondary clusters were also from the southeastern Gansu and adjacent to the primary clusters, which was attributed to the high summer temperature and appropriate rainfall conditions in these regions. These environmental factors were particularly suitable for breeding of mosquitoes, such as *Culex pipiens* and *Culex quinquefasciatus*, resulting in the emergence of a variety of vectors with JEV.^40,41^ In addition, socioeconomic factors may also play an important role in JE transmission. The progress of urbanization is usually accompanied with the decrease of JE incidence over time, when the environment for mosquito proliferation was devastated.^8,12^ Some studies also indicated that rural areas had higher JE incidence rate than urban areas during the study period.^14,37^ Hence, further investigation should be conducted in these high-risk areas to discover the role of biological, social, and environmental factors in the transmission of JE.^42,43^

Currently, JE seems to have a tendency to break through geographical restrictions. Studies had shown that JEV had been found in mosquitoes and pigs in Tibet, and its neutralizing antibodies had also been detected in human serum.^44,45^ Also, geographical expansion of JE cases had been found in Nepal, which mainly expands into hilly and mountain regions.^36,46^ In this study, only one inpouring case of JE was reported in 1981–2016 in the Hexi Corridor, which located in the Western Gansu covering 20 counties. Based on retrospective historical epidemics, 14 counties in the Hexi Corridor region had ever reported clinical diagnosis of JE cases in the 1960s and 1970s, but detailed case information and hospital diagnosis basis could not be found. Therefore, the Hexi Corridor region has been considered as a non-endemic area of JE in Gansu for a long time. However, epidemiological investigations revealed that three JE cases were reported in the Hexi Corridor in 2017, among which two JE cases were probably infected in the other region, and another case without the history of outing was infected in local. Unlike in the southeastern Gansu, owing to only sporadic JE cases in Hexi Corridor and both the children and adult populations may not have been exposed to the JEV, making them susceptible to infection. Hence, we should be aware that the Hexi Corridor may become a new epidemic area of JE, and the prevention and control of JE should be strengthened in this region.

There are some strengths of this study. This is the first study examining spatial and temporal epidemiological dynamics of JE using six decades’ historical data in Gansu, China. It has clearly demonstrated the heterogeneity of JE risk at the county level in Gansu and revealed the spatiotemporal pattern of JE across counties in the province. Moreover, cluster analysis could be an important tool for decision-makers to prioritize areas where more surveillance and disease prevention efforts are required. Our findings can be useful for health authorities to further improve JE control and prevention strategies.

However, several limitations need to be addressed because of the use of historical surveillance data. During the study period 1958–2004, there were no JEV-specific detection methods and techniques such as ELISA and immunofluorescence assay (IFA) applied in Gansu. Therefore, the diagnosis of JE cases was generally based on the epidemiological history and clinical symptoms, lacking confirmation from specific laboratory tests. Thus, there may have underreported JE cases which can influence the quality and sensitivity of JE dataset. In addition, the reported cases were aggregated at the county level, which prohibits analysis at a higher spatial resolution and may lead to important local clusters being missed out. Finally, we only identified potential JE clusters in this preliminary study, but did not explore possible risk factors associated with clustering. Hence, our future research will focus on assessing the association between JE incidence and environmental factors, including climatic, ecologic, and sociodemographic factors, in clustered areas of JE.

## CONCLUSION

In summary, a significant variation in spatiotemporal distribution of JE in Gansu was discovered over the last six decades. This study demonstrates that JE transmission in Gansu was clustered in different spatial and temporal settings, and the geographical distribution of JE appears to have concentrated over the study period. However, there was a rebound of JE incidence in the 2016–2017 after a continuous decrease of JE infection. The geographical range of notified JE cases seems to have significantly expanded in Gansu over recent years. Our findings provided more precise evidence for public health implication on local JE control, particularly for identifying high-risk areas. Based on these findings, further research is needed to explore the impact of socioenvironmental changes (particularly for meteorological and socioeconomic variables) on JE transmission, for establishing locally based early warning system of vector-borne diseases.

## Supplemental figure

Supplemental materials
