# Supplementary figures and images for: Long-Term Epidemiological Dynamics of Japanese Encephalitis Infection in Gansu Province, China: A Spatial and Temporal Analysis
Source: Am J Trop Med Hyg. 2020 Sep 28;103(5):2065–76. doi: 10.4269/ajtmh.20-0179 (PMC7646783; doi:10.4269/ajtmh.20-0179)

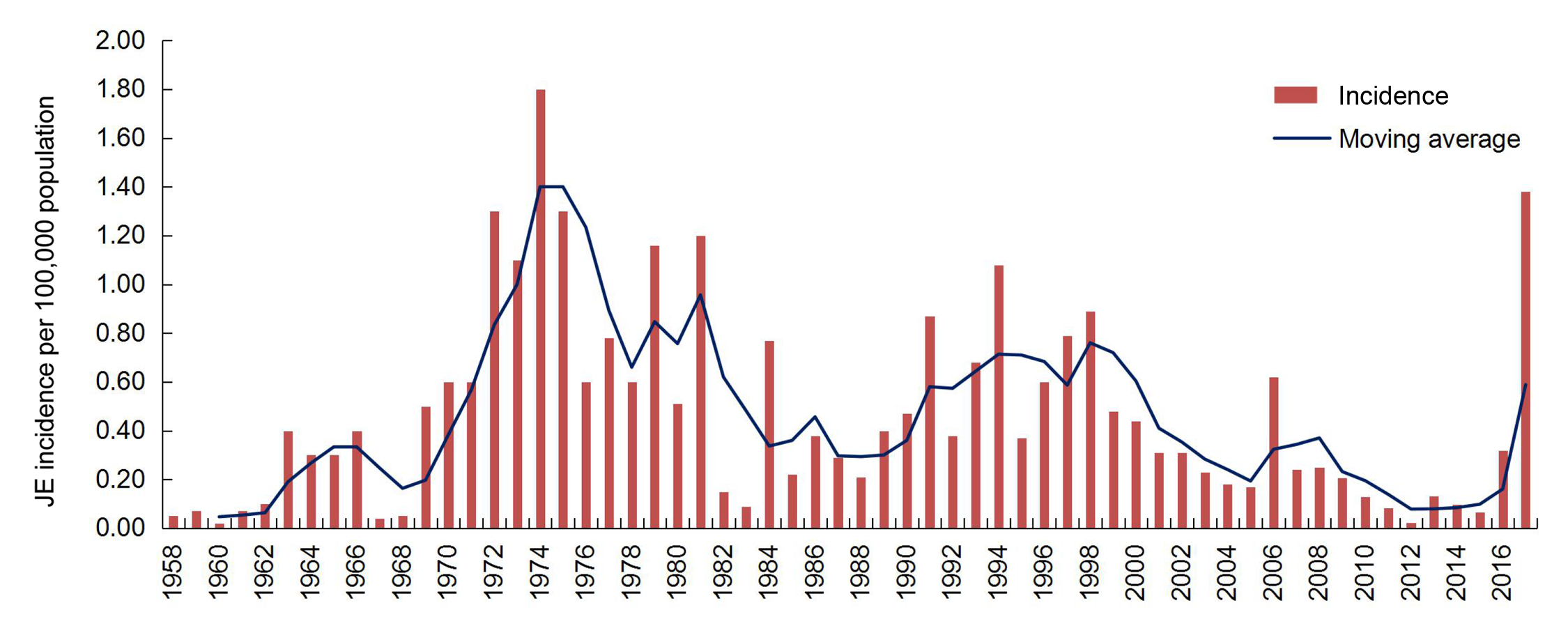

Supplement: Supplementary file 1 [file tpmd200179.SF1.tif]
